# Quantitative comparison of mapping methods between Human and Mammalian Phenotype Ontology

**DOI:** 10.1186/2041-1480-3-S2-S1

**Published:** 2012-09-21

**Authors:** Anika Oellrich, Georgios V Gkoutos, Robert Hoehndorf, Dietrich Rebholz-Schuhmann

**Affiliations:** 1 European Bioinformatics Institute, Wellcome Trust Genome Campus, Hinxton, CB10 1SD, UK; 2 Department of Genetics, University of Cambridge, Downing Street, Cambridge CB2 3EH, UK; 3 Department of Computer Science, University of Aberystwyth, Old College, King Street, SY23 2AX, UK

## Abstract

Researchers use animal studies to better understand human diseases. In recent years, large-scale phenotype studies such as Phenoscape and EuroPhenome have been initiated to identify genetic causes of a species' phenome. Species-specific phenotype ontologies are required to capture and report about all findings and to automatically infer results relevant to human diseases. The integration of the different phenotype ontologies into a coherent framework is necessary to achieve interoperability for cross-species research.

Here, we investigate the quality and completeness of two different methods to align the Human Phenotype Ontology and the Mammalian Phenotype Ontology. The first method combines lexical matching with inference over the ontologies' taxonomic structures, while the second method uses a mapping algorithm based on the formal definitions of the ontologies. Neither method could map all concepts. Despite the formal definitions method provides mappings for more concepts than does the lexical matching method, it does not outperform the lexical matching in a biological use case. Our results suggest that combining both approaches will yield a better mappings in terms of completeness, specificity and application purposes.

## Background

Large-scale mutagenesis projects aim to identify the phenotypes of organisms resulting from modifications to the organisms' genetic markup and thereby provide the tantalizing possibility for revealing valuable information about the molecular mechanisms underlying human disease [1]. In particular, phenotype studies in mice have been demonstrated to provide insights into human disease mechanisms [2], and large phenotype studies are underway with the aim to identify mouse phenotypes resulting from deactivating every single gene in the organism [3,4]. To describe phenotypes within a species and to allow access to the scientific community for further analyses, phenotype ontologies were created to standardize the terminology used in describing phenotypes, e.g. [5,6].

We are now facing the challenge to enable the translation of these species-specific standardized phenotypic information across various species. Two approaches are currently in use for aligning species-specific phenotype ontologies. In the first approach, lexical mappings between the labels of concepts in species-specific phenotype ontologies are used to identify related phenotypes in different species. One implementation of this approach is the Lexical OWL Ontology Matcher (LOOM) [7] which has been shown to perform well on aligning anatomical ontologies. The second approach towards integrating phenotypes across species relies on formal definitions of concepts in phenotype ontologies using the Phenotypic Attribute and Trait Ontology (PATO) [8] and the Entity-Quality (EQ) syntax [9]. The EQ representation allows for the phenotypic definitions to be integrated across species following the application of automated reasoning over their combination with a cross-species anatomy ontology [9,10]. The second approach is implemented in the PhenomeBLAST software [11] and both, software and the resulting mappings, are publicly available from http://phenomeblast.googlecode.com.

It is generally challenging to evaluate and quantify the quality and completeness of ontologies [12]. The challenge is amplified by mappings that involve and bridge multiple ontologies due to the presence of potentially conflicting or implicit conceptualizations by different ontology developers. Furthermore, both the quality of an ontology or of a mapping between ontologies are expected to depend on the specific use-case; ontologies that perform well in one application may not necessarily perform well in other use cases.

Here, we perform a descriptive evaluation of mappings between the Human Phenotype Ontology (HP) [6] and the Mammalian Phenotype Ontology (MP) [5]. We compare the mappings directly and quantify their quality for predicting gene-disease associations based on phenotype data. We find that both methods do not generate a mapping for all ontology concepts and consequently allow for further improvement. Despite the fact that the formal definitions method generates approximately four times more mapped concepts than the lexical matching, it does not outperform the lexical matching in the biological use case. Given the differences in mappings, shown by a deviation when directly comparing the mappings to each other, and availability of mappings with each method, a combination of the results of both methods may lead to mappings which are more comprehensive and specific. The combination may therefore also improve methods that rely on phenotypes for the prioritization of disease gene candidates.

## Results and discussion

### Generated mappings

Table 1 shows the number of mapped concepts available for each ontology and each method. For the formal definitions method, 80% of HP concepts and 50% of MP concepts can be mapped, whereas the lexical matching method provides a mapping for 27% and 12% respectively. Despite the formal definitions method producing a mapping for about four times more concepts than the lexical matching method does, the average amount of mapped concepts to one particular concept is lower. The lower number of mapped concepts for one particular concept suggests that the formal definitions method maps to more generalized concepts (which are higher in the taxonomy) of the other ontology.

**Table 1:** Content of both generated mappings

	HP			MP		
	**HP**	**% total**	**avg # mapped**	**MP**	**% total**	**avg # mapped**

# concepts	10104	100%	-	8507	100%	-
# with formal definition	4860	48.10%	-	5389	63.35%	-
# mapped with lexical	2740	27.12%	7.17	1046	12.30%	6.97
# mapped with ontological	8184	80.10%	5.48	4446	52.26%	6.64

Illustrates the numbers of concepts contained in each ontology but also incorporates the results of the mapping methods. % total: percentages calculated based on the total number of concepts in the ontology; avg # mapped: is the average number of concepts mapped to one particular concept in the ontology.

Both methods are hampered by the definition of concepts in the ontology. The number of mapped concepts and the specificity of the mappings generated by the formal definitions method depends solely on the availability and quality of the formal definitions for both ontologies, which constitutes an advantage at the same time. E.g. a complex phenotypic expression in HP like *Tetralogy of Fallot *which would have no corresponding concept in MP, can still be mapped as long as it is formally defined. The lexical method is limited by the naming of the concepts which is demonstrated by the low number of concepts being mapped from each of the ontologies (four times less than the formal definition method). The number of mapped concepts could potentially be increased by using a less strict text matching algorithm but the method would still rely on the words being used for naming a concept or its synonyms. On average, the method allows for matching more specific concepts than does the formal definitions method indicated by the higher number of mapped concepts from one ontology to the other (see table 1). Given the complexity of some of the phenotypes contained in either ontology, it is still challenging to find appropriate formal definitions in which case the lexical method may align concepts, given that they exist in both ontologies and are defined using the same lexical expression.

### Direct comparison of mappings

**Figure 1:**
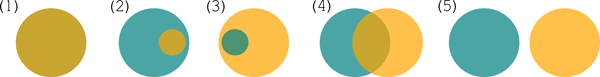
**Overlap groups obtained when comparing both mappings directly**. Shows the different types of obtained overlap while directly comparing the mappings generated by both methods, regardless of the ontology the mapping is provided for. The amount of mapped concepts for the formal definitions method is represented with a yellow circle and the lexical matching is illustrated with a turquoise circle. We identified the following five categories: a) **exact **(both lexical matching and formal definitions method generated exactly the same list of mapped concepts), b) **formal **⊂ **lexical **(mapping generated by the formal definitions method is a subset of the list generated by lexical matching), c) **lexical **⊂ **formal **(mapping generated by lexical matching is a subset of the list generated by the formal definitions method), d) **overlap **(both lists contain additionally mapped concepts and share only a certain overlap), and e) **nothing **(despite both methods generating a list of mapped concepts for a specific concept, both lists have nothing in common).

When comparing the mappings directly to each other, we identified five types of overlap, indicating a deviation in the mappings produced by both the methods. The five different types of overlap are illustrated in Figure 1. The amount of concepts falling into each of the five overlap categories are illustrated in table 2. The table shows that only a low proportion of **exact **matches exists and most of the results fall into the **overlap **category.

**Table 2:** Coverage overlap groups when comparing both mappings

	HP to MP	MP to HP
# exact	155	70
# lexical ⊂ formal	755	287
# formal ⊂ lexical	496	114
# overlap	952	215
# nothing	74	0
# concepts	2432	686

Illustrates the amount of mappings falling into each of the overlap categories when both methods are compared. The mappings for HP to MP and MP to HP are compared independently due to non-symmetrical mappings.

The direct comparison of mappings produced by each method shows that for most of the concepts common to both methods, the mappings share at least some overlap (categories **exact**, ⊂ **formal lexical**, **lexical **⊂ **formal **and **overlap**), even though the number of **exact **matches is low. Four of the categories, **formal **⊂ **lexical**, **lexical **⊂ **formal**, **overlap**, and **nothing **indicate a deviation in both mappings. The category **nothing **points to potential errors in the mappings produced by either method and present a good starting point for further investigations. Once the errors have been eliminated, the distribution of results over all other overlap categories will consequently change.

Given that both methods generate mappings for concepts which are not contained in the other (compare table 1 and 2) and the fact that the results appear as subsets of each other for some concepts (see Figure 1, categories b), c) and d)), it seems to be worthwhile to combine both the approaches and generate one mapping incorporating the results of both methods.

### Impact of mapping methods on biological applications

**Figure 2:**
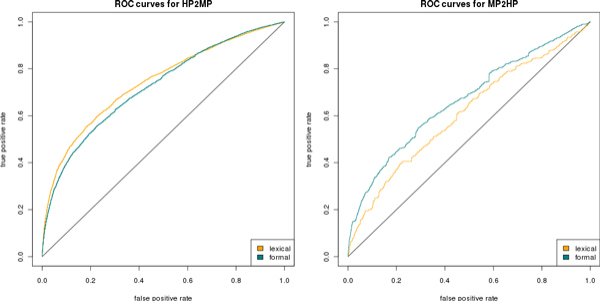
**Receiver Operating Characteristics**. Shows the Receiver Operating Characteristic (ROC) curves for both scenarios: the left panel illustrating the case where alleles are "translated" to HP and the right illustrating the case where diseases are "translated" to MP. In the first scenario the application of the lexical mappings (AUC: 0.74) seems to have better performance than the formal definitions mappings (AUC: 0.72), whereas in the second scenario the formal definitions mappings (AUC: 0.66) seem to yield better results in the biological use case than the lexical mappings (AUC: 0.61).

Figure 2 shows the Receiver Operating Characteristic (ROC) curves for predicting gene-disease associations contained in OMIM's MorbidMap. The true and false positive rates are calculated across all diseases and over all mouse models possessing a phenotype representation compared to the in MorbidMap contained gene-disease associations. We assume that *known *gene-disease associations constitute *positive *examples while *unknown *associations constitute *negative *examples.

The left panel of Figure 2 corresponds to the first scenario in which OMIM diseases are "translated" from HP to MP and the candidate gene prediction is performed by comparing sets of MP concepts. The results show that if the lexical mappings are used, the overall performance for this particular biological use case is better (AUC 0.74) than the mappings generated through automated reasoning (AUC 0.72). The results may be explained with the fact that the HP-based annotations of OMIM diseases use specific ontology concepts (concepts which are deeper in the hierarchy of an ontology). These specific terms (such as *Eosinophilia*) can often be accurately mapped through lexical matching, while a formal definition may not be available due to the complexity of the underlying phenomenon.

The right panel of Figure 2 corresponds to the second scenario in which alleles are "translated" from MP to HP and the candidate gene prediction is performed by comparing sets of HP concepts. The results illustrate that in this particular use case, the application of the formal definitions mappings leads to a better performance (AUC 0.66) than the lexical mappings (AUC: 0.61). Mouse models are less frequently annotated with specific ontology classes that can accurately be mapped through lexical matching. Automated reasoning over the formal definitions provides a sufficient number of mappings for classes that are less specific, while lexical matching does not establish these mappings. Consequently, more information is retained when using ontology-based mappings and the prediction of known gene-disease associations performs better.

## Conclusions

We have evaluated and compared two methods for aligning HP and MP. The first method is based on lexical matching, whereas the second method uses automated reasoning and formal definitions of phenotypes to perform the mapping. While automated reasoning over the formal definitions generates more mappings between both ontologies than lexical matching, these mappings are, on average, less specific than the mappings established through lexical matching. As a result, the mappings perform differently when used for prioritizing disease gene candidates, depending on whether disease phenotypes (which use specific HP phenotypes) are translated into an MP-based representation, or whether MP-based descriptions of mouse genotypes are translated into an HP-based description.

In future research, we intend to extend our analysis of mapping methods and identify strategies to further combine both approaches. Our comparative evaluation can help to improve phenotype-based methods for predicting gene-disease associations and may further extend their capabilities for identifying new gene-disease associations.

## Materials and methods

### Ontological resources

Mammalian Phenotype Ontology (MP): We downloaded an MP version from [13] which was created on the 8th April 2011 and comprised 8,507 concepts. The formal definitions for MP were downloaded separately from the same source. The file provided 5,389 MP concepts with an associated formal definition.

Human Phenotype Ontology (HP): The HP version used for this study, was downloaded from [14]. It was created on the 7th April 2011 and contained 10,104 concepts. The formal definitions were downloaded separately from the same source and provided formal definitions for 4,860 concepts.

### Databases containing gene-disease associations

We used two community-wide established resources containing manually verified gene and disease related data: the Mouse Genome Informatics (MGI) [15] and the Online Mendelian Inheritance in Man (OMIM) [16] database.

The MGI database integrates genetic, genomic and phenotypic information about the laboratory mouse For this study, three of the report files from the MGI database were downloaded [17]

• MGI_GenoDisease.rpt, accessed on 9th March 2011,

• MGI_GenePheno.rpt, accessed on 9th March 2011, and

• HMD_Human5.rpt, also accessed on 9th March 2011.

MGI GenoDisease.rpt contained associations between diseases and specific genotypes (one genotype corresponds to one mouse model) that can be linked to affected genes. MGI_GenePheno.rpt contained the information about genotypes and their observed phenotypes, which are described in MP. HMD_Human5.rpt covered the information about human-mouse orthologous genes.

The OMIM database collects information about human inheritable diseases, including genotype and phenotype information, and known gene-disease associations. It contains about 20,000 entries out of which around 13,000 describe genes and about 7,000 describe diseases. MorbidMap (downloaded on 1st March 2011) contains the up to date information about known links between human diseases and genes. The downloaded version for this study contained 2,717 diseases being linked to 2,266 genes, with 3,463 distinct gene-disease associations. Phenotypic information (HP annotations) for OMIM diseases are available from the HP web page [14]. The downloaded file comprised annotations for approximately 4,000 OMIM entries.

### Mappings between species-specific phenotype ontologies

#### Mappings between ontologies

Let *O*_1 _and *O*_2 _be two ontologies with a set of named concepts *C*(*O*_1_) and *C*(*O*_2_). A mapping between *O*_1 _and *O*_2 _is a set of axioms *Ax *= {*ϕ*_1_(*x*_1_, *y*_1_), ..., *ϕ_n_*(*x_n_*, *y_n_*)} such that *x_i _*∈ *C*(*O*_1_) and *y_j _*∈ *C*(*O*_2_).

Here, we focus on mappings where the axioms relating concepts from two ontologies take the form of sub-class and equivalent-class axioms between atomic concepts. In particular, given the two concepts *A *∈ *O*_1 _and *B *∈ *O*_2_, a mapping involving both *A *and *B *will be of the form

• A SubClassOf: B, or

• B SubClassOf: A, or

• A EquivalentTo: B.

#### Generating mappings through lexical matching

In this study, we used the Lexical OWL Ontology Matcher (LOOM) [7] to generate the lexical matching of concepts between ontologies. LOOM was applied to HP and MP concept names and synonyms. Based on names and synonyms, LOOM extracted 495 HP-MP concept pairs in the form

HP:0002249 MP:0003292.

We imported both ontologies into one single ontology, inserted the pairs extracted by LOOM as equivalence statements and reasoned over the ontology. We generate the mapping by extracting the equivalent and super concepts belonging to the other ontology. In most cases, one concept from one ontology was mapped to multiple concepts from the other ontology.

An example of the resulting mapping looks like

HP:0007062 MP:0000001 MP:0002106 MP:0004142 MP:0004143 MP:0005369.

Due to both ontologies differing in their structure, the mappings are not symmetrical. For example, HP:0008590 'Progressive childhood hearing loss' maps to MP:0006325 'Impaired hearing' but MP:0006325 maps to HP:0000365 'Hearing impairment' (only most specific concepts are given in this example).

The resulting mappings together with the ontology file can be downloaded from the project web page http://code.google.com/p/ontmapcomp/.

#### Mapping through automated reasoning

PhenomeBLAST integrates the formal definitions that were created for classes from the HP and MP [18], including several other ontologies, such as Gene Ontology and UBERON. The ontologies are all converted into OWL EL to enable efficient automated reasoning [19]. PhenomeBLAST then uses the CB reasoner to classify the ontology [20]. To generate the mappings from MP to HP, PhenomeBLAST identifies all equivalent and superclasses of an MP class in HP, and *vice versa *for the direction of HP to MP. The mappings generated by the PhenomeBLAST software are available at http://phenomeblast.googlecode.com and for this study we downloaded the mappings provided (June 2011).

### Direct comparison of mappings

The lexical matching method as well as the formal definitions method generate non-symmetrical mappings for each of the ontologies which results in four mappings in total (compare bottom two rows in table 1). Due to the non-symmetry, the generated mappings had to be investigated independently. For the concepts being represented with either method, we compared the lists of mapped concepts with each other and determined how well the lists overlapped. The direct comparison was executed for both ontologies independently, HP to MP and MP to HP.

### Impact of mapping methods on applications

To assess and quantify the quality of mappings, we additionally used the biological use case of disease candidate gene prioritization to evaluate the performance of each method. For that purpose, we used the phenotype descriptions of mouse models contained in MGI GenePheno.rpt and the OMIM disease HP annotations. Due to the non-symmetry in mappings of either method, we investigated two different scenarios: in the first we "translated" the mouse model MP descriptions to HP using either methods' mapping, whilst for the second we "translated" the OMIM disease HP descriptions to MP. We identified the phenotype similarity between all possible combinations of mouse models and diseases by calculating the phenotype similarity. The phenotype similarity is the cosine similarity between the vector representations of a disease and a mouse model. The cosine similarity is described as:

(1) $$\begin{array}{c} sim\left(A,B\right)=\text{cos}\left(\theta \right)\\ =\frac{A\cdot B}{\left||A||||B|\right|}\\ =\frac{\sum_{i=1}^{n}A_{i}\times B_{i}}{\sqrt{\sum_{i=1}^{n}{\left(A_{i}\right)}^{2}\times \sqrt{\sum_{i=1}^{n}{\left(B_{i}\right)}^{2}}}} \end{array}$$

In the first scenario, both feature vectors are built using MP concepts and in the second, both feature vectors contain HP concepts.

The phenotype similarity score for each disease-model pair was used to rank the mouse models according to their phenotype similarity for each disease. Then, we compared the obtained gene-disease (each mouse model is associated with one gene) pairs to OMIM and recorded the ranks of the known gene-disease associations to evaluate the performance of each method. In the absence of true negative examples, we assume that *known *gene-disease associations constitute *positive *examples while *unknown *associations constitute *negative *examples. The true and false positive rates are calculated across all diseases and over all mouse models possessing a phenotype representation compared to the in MorbidMap contained gene-disease associations. Both true and false positive rates are then used to draw the Receiver Operating Characteristics (ROC) curves (compare Figure 2) for both scenarios of the biological use case.

## Competing interests

The authors declare that they have no competing interests.

## Authors' contributions

AOE designed and executed the study. RH and DRS supervised the work. GG manually validated lexical mappings and investigated the overlap groups resulting from the comparison. All contributed to the manuscript.
